# The content, experiences and outcomes of interventions designed to increase early skin‐to‐skin contact in high‐income settings: A mixed‐methods systematic review

**DOI:** 10.1111/apa.16575

**Published:** 2022-11-03

**Authors:** Chloe Moran, Gill Thomson, Victoria Moran, Victoria Fallon

**Affiliations:** ^1^ Department of Psychology University of Liverpool Liverpool UK; ^2^ MAINN Research Unit, School of Community Health & Midwifery University of Central Lancashire Preston UK; ^3^ Reader in Maternal & Child Nutrition, MAINN Research Unit, School of Community Health & Midwifery University of Central Lancashire Preston UK

**Keywords:** breastfeeding, experiences, intervention, skin‐to‐skin, systematic review

## Abstract

**Aim:**

To explore the content, experiences and outcomes of interventions designed to increase early skin‐to‐skin contact (SSC) in high‐income settings.

**Methods:**

A mixed‐methods systematic review was undertaken across six bibliographic databases. References of all included studies were hand‐searched. All papers were quality appraised using a mixed‐method appraisal tool. A narrative synthesis was used to synthesise both quantitative and qualitative findings.

**Results:**

Database searches generated 1221 hits, and two studies were identified via hand‐searching. Ten studies were included; most (*n* = 7) were designed to improve SSC following a caesarean section, and half were of low/poor quality. Outcomes related to SSC prevalence and/or duration (*n* = 7), breastfeeding prevalence, (*n* = 4) and six explored mothers' and/or health professionals' experiences of the intervention. While the interventions had ‘some’ impact on the prevalence of SSC, the duration was often limited and not in line with WHO recommendations. Breastfeeding rates (exclusive/any) were found to improve but generally not to a significant extent. Mother and healthcare professionals were positive about the interventions, with barriers to implementation noted. Most interventions targeted healthcare professionals, rather than mothers.

**Conclusion:**

High‐quality interventions that increase SSC in line with WHO recommendations, and that target both health professionals and parents are needed.

**Abbreviations**
BFHI: Baby‐Friendly Hospital Initiative
EPIC: Evaluate, Partner, Implement, Consider
ERIC: Expert Recommendations for Implementing Change
i‐PARIHS: integrated‐Promoting Action on Research Implementation in Health Services
MMAT: Mixed‐Methods Appraisal Tool
NICU: Neonatal Intensive Care Unit
PDSA: Plan, Do, Study, Act Model
PEO: Population, Exposure, Outcome
PRECESS: Practice, Reflection, Education and Training, Combined with Ethnography for Sustainable Success
PRISMA: Preferred Reporting Items for Systematic Reviews and Meta Analyses
QI: Quality Improvement
RCT: Randomised Controlled Trial
SSC: Skin‐to‐skin contact
TIDieR: Template for Intervention Description and Replication
WHO: World Health Organisation

**Key Notes**

- Limited improvements in prevalence and duration of skin‐to‐skin were observed, which failed to meet current WHO recommendations.
- No significant improvements in breastfeeding were found.
- Overall, study quality was poor and robust data analysis was lacking.

## 1 INTRODUCTION

Skin‐to‐skin contact (SSC) is a practice where a baby is dried and placed on the mother's bare chest after birth. The World Health Organisation (WHO) defines immediate SSC as beginning less than 10 min after birth and early SSC as beginning any time from delivery to 23 h after birth.^1^ WHO recommends that all mothers and term infants (>37 weeks' gestation) should have a period of uninterrupted SSC immediately after birth for at least an hour or until after the first feed.^1^ The promotion of SSC is in part related to the work of Ann‐Marie Widström. She observed a pattern of innate and instinctive behaviours (subsequently identified and categorised into nine instinctive stages) in babies who were placed SSC with their mothers for an uninterrupted period – referred to as the breast crawl. The nine instinctive stages, the majority of which often occur within the first hour after birth (dependent on infant/maternal health, and the use of analgesia) include: (1) the birth cry, (2) relaxation, (3) awakening, (4) activity, (5) resting, (6) crawling, (7) familiarisation, (8) suckling and (9) sleeping.^2^

SSC has been found to have physiological and psychological benefits for both the mother and her infant.^3^ A Cochrane review of 38 randomised controlled studies involving 3472 women and their healthy, term infants compared early SSC with standard hospital care.^4^ The review revealed that mothers who had SSC were more likely to be breastfeeding at 1–4 months and had breastfed their infants on average over 60 days longer than women receiving standard hospital care. SSC has also been associated with a positive birth experience,^5^, ^6^ reduced incidence of postpartum depression,^7^ and supports maternal–infant bonding.^8^

The Baby‐Friendly Hospital Initiative (BFHI) is a global award designed to improve infant feeding practices; the BFHI is recommended as a minimum standard for maternity services.^9^ One of the criteria for the BFHI award is that newborns are offered a period of uninterrupted SSC immediately after birth for at least an hour or until after the first feed. However, there is evidence to suggest that this is not routinely implemented.^4^, ^10^ Challenges to implementation of SSC include insufficient staffing levels, difficulty identifying eligibility for SSC, safety and practicability concerns,^11^ early transportation of mothers out of the delivery suite, mothers being unwilling to keep their infant SSC,^12^ and lack of knowledge about the benefits of SSC among parents and healthcare professionals.^13^, ^14^ SSC may also be interrupted by others (visitors, family members) wanting to hold the baby post‐birth.^15^ The knowledge and motivation of healthcare staff and parents are also reported to be critical issues in the successful implementation of SSC post‐birth.^16^

A systematic review of 35 studies across 28 countries identified that the prevalence of SSC varied widely from 1% in Tanzania to 98% in Croatia. While higher rates were seen in high‐income countries, there was variability across different settings.^17^ The disparities in rates may be attributed to a lack of consistency in the definition of SSC and in the criteria used to assess the initiation and duration of SSC.^17^ While there is robust evidence for the benefits of SSC on maternal and infant health and wellbeing, there is no comprehensive insight on which SSC interventions, or components of interventions, work best to improve the prevalence of SSC, nor is there synthesised evidence describing the experiences of those receiving and delivering such interventions. This review aims to synthesise the evidence that explores the content, experiences and outcomes of interventions designed to increase SSC in healthy, term infants. As it was recognised that low and middle income countries face different issues in implementing SSC, such as staffing resources, infrastructure and organisation of maternity care, the present review focused on evidence from high‐income settings to allow more comparable findings to be identified.

## 2 METHOD

### 2.1 Search terms and study selection

**FIGURE 1:**
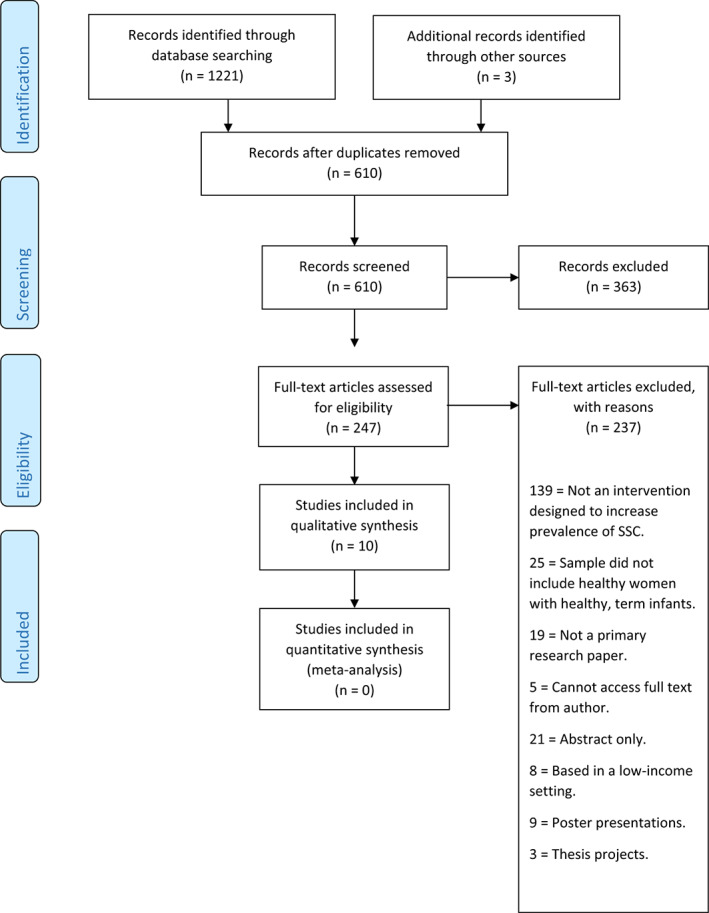
PRISMA flow diagram demonstrating *n* for each stage of the screening process.

A protocol for this systematic review was published in Prospero (#CRD42020168914). A systematic search was conducted across six bibliographic databases in March 2020 and updated in December 2021: Cumulative Index of Nursing and Allied Health Literature (CINAHL), MEDLINE (Ovid), PsycArticles, Scopus, Web of Science and Global Index Medicus, using the search terms presented in Table 1. Search terms were identified using the PEO (Population; Exposure; Outcome) structure. Because definitions of early SSC are wide‐ranging concerning the timing of onset and duration, the search strategy identified all studies related to SSC, which were then screened to identify those that concerned SSC within the first few hours of birth. Figure 1 provides a PRISMA flow diagram displaying the full study selection process.

**TABLE 1:** Search terms

Population	Exposure	Outcome
Women	Education	Skin to skin
Healthcare professional/provider	Intervention	Magic hour
Midwife	Programme	
Mother	Training	
Maternal	Model of care	
Nurse	Implementation	
Lactation consultant		
Doula		
Peer support		
Obstetrician		
Doctor		
Anaesthetist		

Initial title/abstract screening was conducted by one researcher (CM) and checked by another co‐author (VF). Full‐text reviews were then undertaken blind by two authors with the agreement made by consensus. On the occasion that disagreement could not be resolved, decisions about inclusion were made with all four authors. Studies were included if they reported information in a peer‐reviewed journal on the content of intervention(s) designed to increase the prevalence of SSC in the first hours after birth in healthy, term infants in high‐income countries. Studies were excluded based on the following criteria: (a) studies that did not report information on the content of the intervention; (b) low birth weight, very low birth weight, preterm infants, or infants in the NICU, (c) studies conducted in low‐middle income countries, (d) studies that did not specify whether SSC was within the first hours after birth, or (e) results published as conference/meeting abstracts only. No date restrictions were imposed and while searches were conducted in the English language only, no language restrictions were imposed on the search results. References of included studies were hand‐searched to identify relevant studies for inclusion, which led to two additional studies being identified. If additional information was required, that is further methodological detail about the intervention, we attempted to contact the authors directly.

### 2.2 Data extraction

The following information was extracted: bibliographic information, study aims, the definition of SSC used, sample strategy, characteristics and size, study location and context, study design, theoretical underpinnings (if any), the content of the intervention, type of birth, data collection methods and key findings.

### 2.3 Risk of bias (quality) assessment

The Mixed‐Method Appraisal Tool (MMAT) was used to appraise the quality of the included studies.^18^ The MMAT was used as it was designed specifically for use in systematic mixed studies reviews, that is reviews that include qualitative, quantitative and mixed‐methods studies. Two reviewers assessed each included study independently and discrepancies were resolved through discussion.

### 2.4 Data synthesis

As significant heterogeneity meant that meta‐analysis was not possible, narrative synthesis was conducted to synthesise both quantitative and qualitative findings. For qualitative studies, a descriptive synthesis was conducted due to the limited qualitative data included in the studies.^19^ This process involved data familiarisation by reading and rereading the paper, including study results sections and coding the data to identify patterns relevant to the research questions across the studies. Two reviewers (CM and VF) led the data synthesis independently and discrepancies were discussed and resolved. The final synthesis was shared, refined, and agreed with all authors.

## 3 RESULTS

The database searches generated 1221 records, with a further two studies identified through other sources (Figure 1 for PRISMA). Following screening and quality appraisal, 10 studies met the inclusion criteria. A full summary of study characteristics can be found in Table 2. Studies were undertaken in the USA (*n* = 8), Canada (*n* = 1) and the UK (*n* = 1). The included study designs were randomised controlled trial (RCT) (*n* = 1), pre‐post evaluation (*n =* 3), process evaluation (*n* = 1) and quality improvement (*n* = 5). Two of the quality improvement studies complemented their findings with a health records review (*n* = 1) and pre‐post evaluation (*n* = 1). Quality improvement has been described as a combined effort among healthcare staff and stakeholders to diagnose and treat problems in the healthcare system.^20^ The outcomes measured were related to newborn infants (*n* = 4), women (*n* = 9), and health professionals (*n* = 3). Of the studies that reported maternal outcomes, seven were for women who had caesarean sections and three were for those who had vaginal births. Sample sizes ranged from 8 to 1268 participants, but some studies did not specify sample numbers for all outcomes.^13^, ^21^ Outcome measures related to SSC (i.e. prevalence, duration, *n* = 7), breastfeeding (prevalence, *n* = 4) and mothers and/or health professional experiences of the intervention (*n* = 6). The quality of the studies varied, with five of the studies scoring reasonably well,^22^, ^23^, ^24^, ^25^, ^26^ and five scoring relatively poorly.^13^, ^21^, ^27^, ^28^, ^29^ Reasons for poor quality included insufficient detail provided on outcome measures, insufficient attention given to potential confounding factors, and sampling weaknesses. See Table 3 for intervention characteristics.

**TABLE 2:** Study characteristics of included studies (*n* = 10)

							Results		
Study reference	Country	Design	Population	Maternal Age (*M* years)	Sample size	Measures	SSC	BF	Experiences
Boyd^22^	USA	QI	C	NR	*n* = 50 mother‐infant dyads	Questionnaire for nursing staff to complete for each C, collected responses for 1 month. Collected spontaneous feedback regarding participants' observations of the project	37 of 50 mothers and newborns experienced SSC either in the OR or in the PACU. 25 mothers and newborns who experienced SSC did so on the OR bed. The median time newborns spent SSC with their mothers was 42.5 min, exceeding the goal of 15 to 30 min	NR	Qualitative feedback shows patient satisfaction, intervention has continued to attract families to this hospital for their births
Brown, Kaiser, & Nailon^23^	USA	QI	Healthy, term, singleton newborns	NR	Pre‐intervention *n* = 908 Postintervention *n* = 1268	A modified Delphi approach was used to obtain input from stakeholders, who worked through the DMAIC to analyse factors associated with lack of EBF at the hospital and to further identify, plan and implement actions to improve EBF	NR	Chi‐squared analysis revealed a significant difference in number of women EBF in the postintervention period (*p* = 0.001) following clarification of the BF policy and implementation of SSC. In the 7 months prior to initiation of the QI, 55% of all healthy, term singleton newborns (498 of 908) were EBF compared with 64% (812 of 1268; *p* = 0.001) following implementation	NR
Crenshaw et al.^24^	USA	QI Review of electronic health record	V C	29.3	Part 1: *n* = 11 (6:5) Part 2: *n* = 60 (30:30)	Monthly rate of SSC determined using a convenience sample *n* = 60. An advanced practice registered nurse extracted data on 1 day each month from active electronic health records. Excluded if admission documents showed no plans to breastfeed. Noted whether SSC was ‘present‐yes’ or ‘not present‐no.’ Collected the intervention month (August 2010) SSC data after the PRECESS intervention. Rates of EBF based on all babies each month who were eligible for EBF at discharge, one of The Joint Commission Perinatal Care quality performance core measures. A masters‐prepared nurse, knowledgeable about the detailed Joint Commission guidelines, routinely collected these data, noting ‘present‐yes’ or ‘not present‐no.’ Pre‐intervention monthly rates of SSC and EBF at discharge based on rate from July 2010. Postintervention monthly rates of SSC and EBF at discharge based on rates for August–December 2010.	Part 1: 10 (91%) received immediate SSC. Eight (73%) received uninterrupted SSC. Part 2: A significant improvement (25% above baseline) was found in the overall rates of SSC across postintervention months (Pearson v2 = 23.798, *df* = 5, *p* < 0.001), predominantly from improvements in the C population	Part 1: Nine (82%) planned to breastfeed Six (67%) of these babies were EBF at hospital discharge. Five (83%) of the six babies who completed all nine instinctive stages during SSC were EBF at hospital discharge. Part 2: The monthly rate ranged from 49% (*n* = 195 of 397 eligible babies) to 53% (*n* = 184 of 350 eligible babies). Rates of EBF showed no significant change *χ* ^2^ = 2.69, *df* = 5, *p* = 0.748.	NR
Gregson, Meadows, Teakle, & Blacker^25^	England, UK	RCT	C Term (37–42 weeks). Singleton pregnancy. Wish to breastfeed at birth	NR	*n* = 366 (Study = 182; Control = 187)	NR	NR	5% increase in BF rates at 48 h (88% vs 83%) and 7% at 6 weeks (53% vs 46%); however, these differences were not statistically significant (*p* = 0.25 and 0.44). There was a significant correlation between the length of time for which SSC was performed and continuing to breastfeed at 48 h (*p* = 0.04)	NR
Haxton, Doering, Gingras, & Kelly^21^	USA	Pre‐post design	V	NR	Pre‐intervention *n* = 30 5 weeks postintervention *n* = 25 Audit *n* = 30	NR	Pre‐intervention: 30% (*n* = 10) of mothers stated the nurse had discussed SSC before birth, and half (*n* = 15) stated the nurse actually offered SSC. 12 of the 30 mothers said they experienced SSC for between 1–20 min. None of the mothers remained in SSC longer than 20 min, which falls short of the ~1 h needed for a newborn to migrate to the breast. Postintervention: Five weeks after SSC practice was piloted, predischarge interviews with 25 mothers was conducted. 92% reported receiving written information before or after birth from the nurse, and 84% reported that SSC benefits were discussed. 17 (68%) of these 25 mothers experienced SSC for perceived durations of ‘a few’ to 90 min. Eight (32%) of newborns did not receive SSC. A random audit of 30 records was performed at 2 weeks postintervention. All 30 documented SSC education before birth. Seven (23%) did not document any early SSC. Of the 23 remaining charts, six mothers declined SSC and 17 newborns were placed in SSC immediately after birth and remained there for between 5–50 min	In the two quarters since SSC was piloted and then fully implemented, BF initiation rate in term newborns increased from 74% to 84%. Graphs mapped over 12 months prior to and after implementation shows a change from ~76% (quarter 1) to 84% (quarter 3).	Quotes from two mothers who had early SSC anecdotally attest to their positive experience: ‘I forgot about my pain and the baby's colour got better.’ ‘It was wonderful, even though the baby was goopy.’. 1 month after early SSC was piloted, nurses anecdotally reported no increase in workload and no delay in transferring mothers to the postpartum unit and gave the CNS and leadership positive comments about performing SSC with their newborns from mothers.
Hung & Berg^13^	California, USA	Pre‐post design	C Healthy infants	NR	NR	Focussed on the timing of SSC initiation, Latch, Audible swallowing, Type of nipple, Comfort, Hold (LATCH) scores, the use of formula supplementation, the duration of SSC in the OR	<3 months after implementation, the rate of SSC within 90 min from healthy C increased from 20% to 68%, and the rate of infants who did not get any SSC within 4 h decreased from 40% to 9%. <9 months, 60% of healthy infants born by C were SSC in the OR, and 70% were SSC within 90 min of birth.	NR	Positive feedback regarding mothers' experience with SSC in the OR was received. Mothers stated they would like to have SSC with their babies in the OR if they were to experience a C again, and commonly concluded that SSC in the OR made them feel happy.
Turenne, Héon, Aita, Faessler, & Doddridge^27^	Montreal, CA	Pre‐post design	V Nurses	NR	8 births observed pre‐ and postintervention (4:4) 13 nurses observed pre‐ and postintervention (6:7). Survey (*n* = 38)	To guide the development of the intervention, observations of clinical practice was carried out in pre‐intervention, on 2‐day shifts, at the L&D room, to target nurses' learning needs about SSC. An observation grid was used to collect data related to the practice of early SSC. Following the intervention, two evaluations were performed: Nurses' satisfaction regarding the intervention was assessed using a semi‐structured questionnaire. An observation of the clinical practice was also carried out in the L&D room after the intervention to assess the clinical practice related to early SSC. The first author conducted the observation on 2‐day shifts and used the same grid as for the pre‐intervention evaluation.	Seven nurses were observed in immediate postpartum: Four of them received the intervention (two nurses attended two sessions and two nurses attended one session) and three nurses did not receive the intervention. On average, SSC was performed continuously for a period of 26 min. In three of the four cases observed, when SSC was interrupted, newborns were promptly returned on their mother's chest.	NR	For all surveyed nurses, the educational intervention met most of their learning needs and supported the development of their knowledge regarding early SSC. According to them, the duration of the educational sessions was adequate, information was clear, precise, and realistic and teaching/learning methods used encouraged learners' active participation. Main barriers included lack of knowledge among different HCPs, routine care after birth, and resistance to change among HCPs.
Stone, Prater, & Spencer^28^	USA	Process evaluation of an evidence‐based practice change.	C Clinical staff	NR	*n* = 25 for events 1–7 *n* = 25 for events 8–15	All clinical staff who attended the training took a quiz to test their knowledge of the SSC pilot protocol. The protocol was then implemented with a plan to conduct 15 total events of SSC in nonemergent, low‐risk C. The data collection period was 60 days. HCPs were asked to complete a survey after each time SSC occurred in the OR after C. After 7 events occurred, survey responses were compiled and reviewed by the project manager with the champion team to identify any necessary protocol changes. Unfreezing continued with the routine practice of SSC in the OR. Protocol implementation continued until 15 total SSC events were completed.	NR	NR	Most participants agreed or strongly agreed on the ease of use of the protocol. Mean scores for each question increased with subsequent events, indicating the staff members were more comfortable in their roles and with the protocol. Responses implied more comfort with the process and support of the protocol as noted by specific statements made on the questionnaire, such as ‘none’ to the question about obstacles and barriers, and ‘went smoothly and did not interfere with my job’, and ‘very important for patient satisfaction, I'm glad we are implementing this’.
Thompson & Maeder^26^	USA	QI Pre‐post design.	C	NR	*n* = 40	Measured nurses' knowledge and self‐reported SSC practices with pre‐implementation and post‐implementation surveys. Measured nurses' inclusion of SSC in the interdisciplinary TTO and actual SSC practices in the OR with an audit tool.	In the pre‐implementation phase, SSC was included for 70.9% (56/79) of eligible C's, and in the post‐implementation phase, it was included for 75.3% (70/93). SSC in the OR for 15 min increased from 20.3% (16/79) in the pre‐implementation phase to 27.5% (61/222) in the implementation phase but decreased to 24.7% (23/93) in the post‐implementation phase. However, a chi‐square analysis showed an overall statistically significant difference in SSC for less than 15 min (partial SSC) performed in the OR across the pre‐implementation, implementation, and post‐implementation periods (p < 0.001). SSC for less than 15 min increased from a 10.1% of the time before implementation (8/79 observations) to 36.6% of the time after implementation (34/93 observations). Reports of no SSC in the OR decreased from a reported 69.6% of the time pre‐implementation (55/79 observations) to 38.7% of the time post‐implementation (36/93 observations). Total SSC for any length of time, including SSC for 15 min or more and for less than 15 min, increased throughout the initiative. Total SSC was 30.4% (24/79) pre‐implementation (20.3% [16/79] for 15 min or more and 10.1% [8/79] for less than 15 min) to 61.3% (57/93) post‐implementation (24.7% [23/93] for 15 min or more and 36.6% [34/93] for less than 15 min)	NR	NR
Grassley & Jones^29^	USA	QI	C	NR	44 families	After each birth, the nurses recorded minutes of SSC, barriers encountered, and their perceptions of the experience for themselves and their patients on an outcomes form, which were analysed for frequency of SSC in the OR, types of barriers, and nurse reports of family responses.	80% held their newborns SSC in the OR, 43% for a minimum of 15 min, and 37% for less than 15 min. The other 20% declined (11%) or were unable to hold their newborns due to an infant or maternal condition (9%). Barriers included difficulty positioning the newborn related to size or positioning of the surgical drape, mothers feeling nauseous or claustrophobic, infant condition, and short length of surgery.	NR	87% of nurses reported feeling prepared to assist with SSC in the OR. SSC in the OR appeared to facilitate a positive birth experience. The nurses repeatedly reported observing a positive response by mothers and their support persons to the experience of holding their newborns SSC, particularly women who had had previous C.

Abbreviations: BF, breastfeeding; C, caesarean births; E, experimental; EBF, exclusive breastfeeding; HCP, healthcare professional; L&D, labour and delivery; *M*, mean; NR, not reported; OR, operation room; PACU, post‐anaesthesia care unit; QI, quality improvement project; RCT, randomised controlled trial; SSC, skin‐to‐skin contact; TTO, team time‐out; V, vaginal births.

**TABLE 3:** Intervention characteristics

Study reference	Aims	Theoretical underpinnings	Definitions and timings of SSC/intervention	Content of intervention	Mode of delivery
Boyd^22^	Provide the learner with knowledge of best practices related to initiating SSC between a mother and newborn during and after C. Implement initiation of SSC between mother and newborn within 1 h of C.	The Iowa Model of Evidence‐Based Practice.^30^	SSC to occur immediately following C, in the OR or PACU (*n* = 37) or on the OR bed (*n* = 25).	Developed SoP for SSC in the OR based on available evidence. Information and key literature were shared with staff to support and explain risks and benefits of SSC. For all who qualify to experience SSC, the baby nurse will perform the following interventions: Place a warming blanket on the mother at the start of the C. Loosen the mother's gown after she is placed on the OR bed in anticipation of SSC. Release the mother's arms from straps on arm boards. Place the newborn, wearing only a diaper, on the mother's skin on the OR bed. Cover the mother and the newborn with a warming blanket. Encourage the significant other to touch and support the newborn. Have the significant other hold the newborn during maternal transfer from the OR bed to the stretcher. Transport the newborn and the mother to the PACU on the stretcher, maintaining SSC. If the mother is unable to hold the newborn, escort the significant other to the PACU for SSC with the newborn. Continue SSC in the PACU. Delay instillation of the newborns eye ointment and vitamin K injection until arrival in the PACU. The baby nurse will initiate or continue SSC in the PACU by: Continue SSC as in the OR. If the mother is unable to hold the newborn, offer SSC to the significant other until the mother is stable. Initiate standard care for the newborn after delivery according to facility protocol. Initiate standard care for the mother after C, according to facility protocol. Initiate BF for the mother and the newborn. All personnel with potential to be involved in implementing the practice change received letters explaining proposal and its purpose, lists of contact staff members for questions or problems, and copies of the SoP. An implementation committee was also recruited to disseminate information about the proposed change and to have champions available at all times to answer questions. After SoP was approved, it was incorporated into the hospital's online practice standards where it was accessible to all staff members. Ten days before implementing SSC in the OR, an email was sent to all team members reminding them of the implementation date for the practice change. An informational handout was developed for parents regarding SSC, and key stakeholders reviewed it before use. This handout was placed in the admission packet, which was given to all mothers to educate them regarding SSC.	Face‐to‐face Online Materials: Staff letters List of staff contacts Copies of SoP Email reminder Informational handout
Brown et al.^23^	To increase the per cent of healthy, term singleton newborns who were exclusively breastfed.	Six Sigma Define, Measure, Analyse, Improve, and Control (DMAIC) quality improvement methodology.^32^ Translational research model.^33^	Defined as immediate, uninterrupted SSC until after the first BF episode. Project was conducted over a 12‐month period.	Actions involved clarification of the existing BF policy and provider orders, explaining that nurses were not to supplement breastfed infants without provider order. An evidence‐based practice of SSC at birth was identified. Implementation of SSC at birth began with initial dissemination of the evidence on SSC provided to nurses and SSC measured through qualitative interviews conducted with BF mothers on the first or second postpartum day. An understanding of the advantages of EBF and associated interventions such as SSC were communicated with nursing staff including electronic media via e‐mails with links to You‐Tube videos, web resources, and related research abstracts; discussions and presentations at unit meetings; bulletin board postings; an optional 4‐h didactic class; and interaction with change champions and opinion leaders. Change champions and opinion leaders attempted to provide one‐on‐one discussions with nurses who were not yet practicing SSC to identify barriers, answer questions, and reinforce the rationale for the practice change. A meeting was held with obstetric residents who attend the majority of deliveries to communicate the QI and discuss their role in the implementation of SSC. To enhance the structures supporting EBF, the organisation's BF policy was revised to add the expectation of SSC at birth and to provide clarity on supplementation orders. Retrospective audits/evaluation of qualitative interview data from women was undertaken, with weekly emails sent to all nursing staff recognising those who had two or more V deliveries of term newborns and had achieved greater than 50% SSC.	Face‐to‐face: Staff discussions Interactions with change champions and opinion leaders Optional classes Online: Emails Identification and praise of staff achieving >50% SSC YouTube Web resources Presentations Bulletin board postings
Crenshaw et al.^24^	To improve skin‐to‐skin care and EBF at hospital discharge.	PRECESS Model.^34^	SSC to occur immediately after birth, 5‐day intervention period.	Invited various staff groups to optional formal educational sessions about the evidence in support of skin‐to‐skin care, the process of clinical care during skin‐to‐skin care after vaginal birth and during and after caesarean surgery, and the newborn's nine instinctive stages during SSC. None of the physicians or nurse anaesthetists (Certified Registered Nurse Anaesthetists) attended the formal sessions. Between formal training sessions, the principal investigator and PRECESS team were present on the unit. Hospital team members encouraged clinicians and staff to support their patients' participation, obtained consents from patients, provided clinical care during SSC, and coached their colleagues in providing SSC and overcoming potential barriers. Distributed tear sheets to clinicians, staff, and parents that illustrated a newborn's nine instinctive stages during SSC in the first hour after birth. Waited until birth was imminent and then entered the LDR or OR. Mothers and babies were recorded with a video‐camera during SSC, began at birth and continued during SSC period. Discontinued video‐recording if a complication occurred and resumed with problem resolution. Direct care nurses dried the babies on the mother's abdomen with warmed blankets, placed them SSC on the mother's chest, covered them with dry warmed blankets, and placed a cap on the head of some babies. Babies born by caesarean surgery were either dried with warmed blankets on the mother's chest, above the sterile field, or dried with warmed blankets in a cart as babies were moved from the surgical site to the mother's chest. The staff did routine assessment and care procedures during SSC. PRECESS experts and study team verbally coached clinicians, staff, and families during SSC. The video‐ethnographer edited the video‐recordings daily and labelled newborn's instinctive stages and the minutes after birth that the stage occurred. Used an LDR next to the nurses' station for informal education and to provide opportunities for clinicians and staff to analyse and reflect upon video‐recordings. Compiled strategies identified by hospital staff to implement SSC	Face‐to‐face: Optional educational sessions Verbal coaching during SSC Video‐recordings of SSC with opportunity for staff to watch, reflect upon and analyse Materials: Tear sheets to staff describing nine instinctive stages
Gregson et al.^25^	To determine whether kangaroo care (SSC) between mother and baby in the OR can affect BF outcomes following an elective C.	NR	SSC to occur immediately after birth. Encouraged to maintain SSC as much as possible within the first 48 h of life.	Participants in the study group received a KangaWrap Kardi to wear underneath the operation gown, prior to admission to theatre. The garment had been designed and developed to help women confined to bed (e.g., following C) to perform SSC with their baby. At birth, the baby was placed SSC prone on the mother's chest. Training sessions were held with staff in the OR to ensure they were comfortable with tying the KangaWrap Kardi, positioning of the mother with one pillow during the operation and also to clarify that the midwife was responsible for observing the baby while in the operating theatre so that any deterioration in the baby's condition would be observed and acted on immediately. Participants in the control arm of the study received ‘normal care’ as follows: following the birth the baby, it was placed in its mother's or father's arms and parents were encouraged to have at least 1 h SSC.	Face‐to‐face: Training sessions with staff covering use of equipment
Haxton et al.^21^	This article provides information about our process and challenges of implementing SSC.	Iowa Model of Evidence‐Based Practice.^30^	Aimed for SSC to be performed for a minimum of 1 h immediately after birth. Intervention spanned 6 quarters from 2010 (Q1‐Q3) to 2011 (Q1‐Q3).	The CNS delivered four educational sessions for the L&D nurses. Delivered via PowerPoint and DVD presentations to illustrate the importance of early SSC and give practical advice for nurses. Nurses were also given the opportunity to discuss their concerns about possible barriers and solutions to implementing SSC. Prior to implementing SSC, each mother received written information regarding the benefits of SSC. With the mother's consent, the intervention consisted of immediately placing the newborn on a warm blanket on the mother's abdomen and allowing the newborn to rest until the cord was clamped and cut. The initial blanket would be removed, and the newborn would be placed directly in SSC on the mother's chest. A diaper and cap would be placed on the newborn, and both mother and newborn would be covered with a warm blanket. The initial newborn assessment, assignment of Apgar scores and placement of ID bands would occur while the newborn was in SSC. If providers or parents wished to have the newborn weighed for documentation purposes, this may be done quickly, with the newborn being immediately returned to SSC. L&D nurses would remain available to assist and coach the mother with positioning for BF but would generally try to allow the baby to move spontaneously toward the breast. Mothers who chose to formula feed or delay initiation of BF would still be encouraged to perform immediate SSC, so their babies could receive the non‐BF‐related SSC benefits. Any mother who did not want to perform SSC would be given the newborn after drying and warming, as was done previously. A brochure was created outlining the benefits of SSC, including an illustration of a newborn in SSC. This educational tool would be provided to mothers on admission to the unit to give time to decide whether they would like to engage in SSC.	Face‐to‐face Technology: PowerPoint presentation DVDs Materials: SSC brochure provided to mothers on admission
Hung & Berg^13^	To increase the rate of early SSC among healthy infants born by C.	The Plan‐Do‐Study‐Act Model.^31^	SSC initiated immediately after birth in OR for ≥15 min	During planning phase, surveyed the nursing staff to gather ideas on the barriers and solutions to implementation, and to find the nurses interested in becoming champions of the improvement project. Visited the other Baby‐Friendly hospital in the area for practical insight. Reviewed the literature and consulted with key stakeholders. Then drafted a flowchart to outline the team‐based intervention process. The final decision regarding the appropriateness of SSC in the OR was left to the discretion of the onsite team after the newborn was initially assessed and dried. If eligible for SSC in the OR, the nursery charge nurse supported SSC for 15 min, or however long the mother was able/willing. The blue drape, which provided a barrier between the mother's upper body and the lower sterile field, was hung below the mother's breasts, allowing space for the infant to lie across the mother's upper chest. The nursery charge nurse assessed the spatial setup and I.V. placement, untied the mother's arms, lowered the mother's gown, placed the naked infant transversely across the mother's bare chest covered by warm blankets, and placed a cap on the infant's head. All staff members were encouraged to participate in implementing the intervention and continued collecting data to monitor improvement. Piloted several cases with scheduled caesareans and modified flowchart with feedback from the nurses. For the first few cases, the Perinatal CNS remained in the OR and assisted the nursery nurse in positioning the infant. Then offered in‐service education to the nursing staff and paediatric residents and posted a bulletin board presentation in the birth centre and the nursery that was visible to both staff and patients. Distributed the flowchart to the staff and posted it in the OR. Kept staff up‐to‐date on progress by posting result data and patient feedback.	Face‐to‐face: In‐service education to the nursing staff and paediatric residents. Materials: Posted a bulletin board presentation in the birth centre and the nursery that was visible to both staff and patients. Flow chart distributed to staff and posted in OR.
Turenne et al.^27^	Highlight the implementation of educational intervention aimed at an evidence‐based practice change regarding early SSC in a maternity unit.	The Iowa Model of Evidence‐Based Practice.^30^	Planned timings of SSC NR. Educational intervention conducted over a 1 month period (Jun‐Jul 2013)	Four 30‐min educational sessions delivered by nurses to develop their knowledge and skills about SSC promptly after birth. Sessions were given twice on lunch break and offered about a week apart, allowing for the application of new knowledge into practice. Sessions took the form of small working groups, where interactions between the participants was encouraged. Session 1: Definition of SSC, benefits (mother and newborn), possible solutions to enhance actual practice. Via lecture, group discussion, short video, and individual reflection. Session 2: Safe installation of the newborn in SSC, guidelines for the practice of SSC in immediate postpartum. Via role‐playing, scenario, group discussion, and lecture. Session 3: Assessment of couple's expectations regarding childbirth, information to give couples on progress of childbirth and interventions in immediate postpartum. Couple's education (SSC benefits), proper monitoring during SSC. Via simulations and group discussion. Session 4: SSC with premature and/or low birth weight infants. SSC post‐C. Via quiz and lecture.	Face‐to‐face: Lectures Discussion in large groups Individual reflection Role‐playing ‐ demonstration of SSC installation. Scenarios Simulations Media: Short videos Materials: Quiz List of SSC benefits displayed around the maternity unit and a copy for each participant. Two scientific articles on SSC with abstracts offered. Five posters in childbirth rooms extolling the benefits of SSC.
Stone et al.^28^	To develop a protocol for HCPs roles in providing SSC in the OR. To implement the protocol. To evaluate the process of implementation of the evidence‐based intervention.	Iowa Model of Evidence‐Based Practice.^30^ Lewin's Change Theory.^45^	The newborn would be brought to the mother for SSC at 5 min of birth if condition was stable. Data collection was completed over 60 days.	The project manager and nurse manager identified champion representatives from several disciplines that are present during C. These representatives were charged with collaborating as a team to communicate concerns regarding SSC in the OR after C. After compiling concerns from all champion team members, the project manager designed and led the team in a simulation experience in the OR to develop a preliminary protocol for SSC after C. Once the champion team members were identified and support from physicians was obtained, the next step was to coordinate and lead a 1‐h OR simulation with the identified champion team. Prior to the simulation, the obstetrician, anaesthesia provider and lactation consultant verbally shared their opinions and their full support for SSC was conveyed to the project manager. Their input was incorporated during the simulation. The project manager conducted multiple educational sessions for nursing staff, obstetricians, and anaesthesia personnel regarding the benefits of SSC and implementation of the SSC after C pilot protocol. All personnel who attend C received a training session on the SSC protocol, completed a quiz and signed their consent. Not all staff members attended the face‐to‐face educational sessions; however, anyone assigned to the OR was required to read the protocol, complete the quiz, sign the consent, and verbalise their understanding before participating in the SSC project. Following the educational piece of the project, the pilot protocol was implemented on a trial basis.	Face‐to‐face educational sessions. Materials: Post‐educational test about SSC in the OR.
Thompson & Maeder^26^	To increase nurses' knowledge of SSC benefits immediately after birth to increase nurse‐reported SSC practices in the OR. To increase communication among the interdisciplinary team members and women about the benefits of SSC in the OR by including SSC as a part of the TTO that is verbalised before every C. To increase the percentage of eligible women and newborns who undergo SSC in the OR to 50% in the post‐implementation phase.	NR	SSC delivered immediately after C, in the OR, for at least 15 min within the first hour of life. Intervention period spanned 4 months (Feb‐May)	(1) Provided educational posters throughout the unit and weekly e‐mail reminders of the benefits of SSC to educate nurses. Educational posters included recommendations for SSC according to the AAP, WHO, and Centres for Disease Control and Prevention, as well as strategies to mitigate barriers to SSC in the OR. Also presented these posters during daily safety huddles to include the interdisciplinary team. One of the posters also remained on display in the main hallway to the OR so that all staff members could view it regularly. Modified the curricula for the new nurse orientation training and the circulator nurse training to include SSC in the OR as part of the expected care for eligible women having C and their newborns. Finally, we made corresponding modifications to the information provided to parents who attend a hospital tour before birth to include information about SSC in the OR. (2) Aimed to modify the structure of the TTO specifically for initiative purposes, which is a safety check completed before the beginning of any C procedure. Added SSC to the standard TTO template structure so that the interdisciplinary team could identify possible contraindications to performing SSC or anticipate initiating SSC in the OR. Placed printed TTO posters in each of the four ORs as a visual reference for the team to include plans for SSC in the TTO for every C. (3) Created a new practice guideline to include SSC in the OR for at least 15 min within the first hour of life for all eligible newborns after C	Face‐to‐face meetings Presentations during huddles and interdisciplinary meetings Training courses to implement new curriculum Materials: Benefits and recommendations poster in the hallway Strategies to combat barriers to SSC flipboard in the OR ‘10 Tips for Successful SSC in the OR’ poster Weekly email reminders of the benefits of SSC throughout implementation Pre‐ and post‐implementation surveys emailed to staff
Grassley & Jones^29^	To evaluate the feasibility of implementing SSC in the OR.	NR	SSC within 45 min of C, infant was firstly dried, assessed, then placed SSC with blanket on back.	First formed an interprofessional team who met regularly to strategize implementing the practice change. To plan a feasible process that addressed potential barriers and staff concerns about implementing SSC in the OR, the project team met in one of the birthing unit ORs to problem‐solve surgical team roles and environmental logistics. Final step was to practice protocol with team members enacting the various roles (including the mother) until they had developed the best process. To facilitate staff preparation, collaborated with a group of nursing students from academic partner university, who developed a learning module that included a PowerPoint and companion video. The students obtained permission to film a simulation of the protocol in the hospital OR, using themselves as the actors and props provided by the L&D unit educator for a realistic C setting. The video was edited to include voice‐over narration. The video and PowerPoint were uploaded onto the unit computers and watched by the staff. The students also developed a parent flyer that included information about SSC in the OR and was distributed at the partner physician group office. After receiving a determination of exemption from the hospital's institutional ethics review board, they piloted the protocol with patients from their physician partner group who were scheduled for a repeat C. Families were asked upon admission to L&D if they would like to participate. If they answered yes, the OR team was informed, and the protocol followed. Goal for SSC in the OR was a minimum of 15 min because this mirrored the average length of surgery for many physicians.	Materials: PowerPoint presentation Parent flyer Media: Videos

Abbreviations: AABM, American Academy of Breastfeeding Medicine; AAP, American Academy of Paediatrics; AWHONN, Association of Women's Health, Obstetric and Neonatal Nurses; BF, breastfeeding; C, caesarean birth; CNS, clinical nurse specialist; HCP, healthcare professionals; L&D, labour and delivery; NR, not reported; OR, operating room; PACU, post‐anaesthesia care unit; QI, quality improvement project; SoP, standards of practice; SSC, skin‐to‐skin contact; TTO, team time‐out; V, vaginal birth.

### 3.1 Narrative synthesis of quantitative data

Four quality improvement (QI) projects were conducted in the USA.^22^, ^24^, ^26^, ^29^ Boyd^22^ examined 50 mother‐infant dyads born by caesarean section, of which 37 experienced SSC. Using the Iowa Model of Evidence‐Based Practice to Improve Quality of Care,^30^ they developed SSC standards of practice in the operating room (OR) and shared information with staff to support and explain the benefits of SSC. After implementation of the SSC standards, they found that the median time that the infants spent in SSC were 42.5 min which exceeded their original goal of 15–30 min. However, as no baseline median of SSC was reported it is unclear whether SSC rates exceeded pre‐intervention rates. Similarly, Grassley and Jones^29^ developed a protocol to implement SSC following repeat caesarean birth. This involved a training module comprising a PowerPoint presentation and companion video simulation for OR staff. Here, 80% of the 44 included families held their newborns in SSC in the OR immediately after birth, with 43% experiencing 15 min or more of SSC. Crenshaw et al.^24^ used the PRECESS approach (Practice, Reflection, Education and training, Combined with Ethnography for Sustainable Success) during a 5‐day quality improvement study with infants born vaginally and by caesarean section and compared rates of SSC pre and post‐intervention. The first stage of the intervention involved video ethnography and interaction analysis of 18 mothers and found that 10 received immediate SSC, while 8 received uninterrupted SSC. The second stage examined electronic health records of 60 women (*n* = 30 vaginal birth; *n* = 30 caesarean section) to compare differences in SSC rates and found a significant 25% improvement in overall rates of SSC post‐intervention (*χ*[2] = 23.798, *df* = 5, *p* < 0.001). Improvements in SSC were predominantly seen in those women who had caesarean sections. Thompson and Maeder^26^ utilised educational posters and weekly email reminders featuring benefits and recommendations for SSC to aid staff education. Practice guidelines were updated, team time‐outs were restructured, and orientation training was adapted to include SSC in the OR as part of expected care for women delivering via caesarean section. Overall, total SSC maintained for any length of time was seen to increase throughout the project (*n* = 40). A chi‐squared analysis revealed a significant increase in SSC for less than 15 min across all three phases (pre‐implementation, implementation, post‐implementation). SSC in the OR for 15 min increased from 20.3% at pre‐implementation to 27.5% in the implementation phase but reduced to 24.7% post‐implementation (*p* < 0.001). Reports of no SSC decreased from 69.6% pre‐implementation to 38.7% of the time post‐implementation (*p* < 0.001).

Three pre‐post intervention studies were conducted in the USA and Canada. Haxton et al.^21^ developed and implemented a SSC protocol using the Iowa Model,^30^ in a labour and delivery unit in the USA. This was a seven‐step intervention which included the development of a step‐by‐step protocol and a patient education brochure. Pre‐intervention, interviews were conducted with 30 mothers who gave birth vaginally to healthy, term newborns to explore their experience of SSC. A third of mothers (*n* = 10) stated the nurse had discussed SSC before birth and half (*n* = 15) stated the nurse had offered SSC. Twelve (40%) mothers had experienced SSC for between 1–20 min, but none remained in SSC longer than 20 min. The intervention was then introduced that involved staff being educated on the protocol and with modifications made as needed. An audit conducted 2 weeks post‐intervention found that 100% of 30 randomly selected medical records documented that SSC education had taken place before birth. All mothers that agreed to SSC had records indicating that this occurred immediately after birth and for between 5–50 min. Five weeks after the intervention was delivered, interviews with 25 mothers revealed that 92% reported receiving written information before or after birth from the nurse and 84% reported that SSC benefits were discussed. Around two‐thirds of mothers (*n* = 17) reported that they had SSC for periods ranging from ‘a few minutes’ to 90 min, and approximately a third of mothers (*n* = 8) stated that they had not received SSC.

Turenne et al.^27^ also used the Iowa model^30^ to develop and evaluate an educational intervention to improve SSC in a maternity unit in Canada. Four 30‐min educational sessions were delivered to nurses in small working groups to develop knowledge and skills about SSC. Pre‐intervention, four women who had vaginal births and six nurses were observed and a mean SSC length of 20 min was recorded. Post‐intervention, observations of four women who had vaginal births and seven nurses revealed an increased mean SSC length of 26 min. The limited impact of the intervention may be due to a lack of engagement with the educational sessions, as none of the nurses had attended all four sessions and three had not attended any.

Hung & Berg^13^ used the Plan‐Do‐Study‐Act Model (PDSA)^31^ to increase the rate of early SSC among healthy infants born by caesarean, both in the OR and during recovery in a birth centre in the USA. They developed and distributed a flowchart to outline the intervention to OR staff, provided in‐service education to nursing staff, and posted a bulletin board presentation that was accessible to staff and women. During the first 3 months after implementation, SSC rates occurring within 90 min from birth increased from 20% to 68% (*n* not stated). The prevalence of infants who did not get any SSC within 4 h decreased from 40% to 9%. Within 9 months of the intervention, 60% of infants born by caesarean received SSC in the OR and 70% received SSC within 90 min of birth.

### 3.2 Effectiveness and experiences of interventions for improving breastfeeding outcomes

Overall, four SSC intervention studies were designed to improve breastfeeding rates. Three were undertaken in the USA and one in the UK. In the QI study by Brown et al.^23^ they used the Six Sigma Define, Measure, Analyse, Improve and Control (DMAIC)^32^ QI methodology and Titler and Everett's Translational Research Model.^33^ A modified Delphi approach was also used to obtain input from stakeholder groups to identify, plan and implement an evidence‐based practice of SSC at birth. The aim was to improve exclusive breastfeeding rates of healthy, term, singleton births during infant hospitalisation in the US. A pre‐post‐cohort study was undertaken and found that while 55% of newborns (498/908) were exclusively breastfeeding in the 7 months before implementation, this figure significantly increased to 64% (812/1268; *p* = 0.001) following implementation.

Likewise, Crenshaw et al.^24^ conducted a similar QI project also based in the USA using the PRECESS model described above.^34^ A pre‐post cohort design was used and a convenience sample (that involved reviewing medical records) of women who had given birth to a healthy infant (vaginally or via caesarean) and were eligible for exclusive breastfeeding. The study found no significant difference in exclusive breastfeeding rates at hospital discharge across the cohort (monthly rate ranged from 195/397 (48%) to 184/350 (53%); Pearson *χ* ^2^ = 2.690, *df* = 5, *p* = 0.748).

Haxton et al.^21^ aimed to increase breastfeeding initiation rates by implementing SSC practices using the Iowa Model in a US hospital. The intervention involved disseminating a brochure to mothers upon admission to the unit outlining the benefits of SSC along with an illustration of a newborn in SSC. In the 6 months following the introduction of the intervention, breastfeeding initiation rates in term newborns increased from 74% to 84% (*n* not stated).

Gregson et al.^25^ carried out a RCT of 366 term singleton births in the UK to determine whether the introduction of SSC in the OR affected breastfeeding outcomes following an elective caesarean section. Mothers in the experimental group were asked to wear a KangaWrap Kardi designed to help women confined to bed perform SSC with their infants. At birth, participants were encouraged to maintain SSC as much as possible during the first 48 h of life. Training sessions were also held with staff in the OR. Participants in the control arm received ‘normal care’ as standard. This involved the baby being placed in its mother's or father's arms following the birth and parents being encouraged to have at least 1 h SSC. Overall, there was a 5% increase in breastfeeding rates at 48 h (88% vs 83%) and a 7% increase at 6 weeks (53% vs 46%) in the intervention arm; however, these differences were not statistically significant. In contrast, there was a significant correlation observed between the length of time SSC was performed and continuing to breastfeed at 48 h (*p* = 0.04, Fisher's test statistic not reported).

#### 3.2.1 Experiences of SSC interventions

Five of the included studies also explored mothers' and/or health care professionals' experiences of the interventions. Two studies focused on the experiences of health care professionals using a mix of quantitative and qualitative questions,^27^, ^28^ and three studies collected both staff and mother's experiences using qualitative feedback.^13^, ^21^, ^22^

#### 3.2.2 Staff experiences

Two studies explored staff satisfaction with the SSC intervention in the labour and delivery room. Turenne et al.^27^ used a survey to capture 20 nurses' (from a potential sample of 50) satisfaction with the intervention. Overall, the participants reported a high level of satisfaction with the intervention content (*n* = 19), modality (*n* = 18), and duration (*n* = 18), as well as the teaching/learning methods used (*n* = 20). The main facilitator of SSC was perceived to be continuing education for healthcare professionals (*n* = 11). Whereas the most common barrier was the lack of knowledge among different health care professionals (*n* = 8). Haxton et al.^21^ reported that 1 month after early SSC was piloted, nurses anecdotally reported no increase in workload or delays in transferring mothers to the postpartum unit and that positive comments had been received from mothers about performing SSC with their infants.

Hung and Berg^13^ provided only sparse data on ‘informal responses’ from staff who gave ‘positive responses’ after receiving the in‐service education on the benefits of SSC after caesarean section and that they ‘understood the need for improvement’ (p. 323). Two studies more formally explored staff satisfaction with implementing SSC in the OR after a caesarean section. Stone et al.^28^ used surveys to collect staff views after 15 episodes of implementing SSC to capture whether the protocol was followed, team coordination, comfort in role and ease of protocol. Overall, 36 surveys were collected. The findings indicated that staff members were more comfortable in their roles and with the protocol over time. Staff could also provide free‐text comments on the survey, with comments such as the SSC intervention ‘went smoothly and did not interfere with my job’ and how SSC was ‘very important for patient satisfaction, I'm glad we are implementing this’, being recorded. Boyd^22^ recorded spontaneous verbal comments regards the implementation of SSC in the OR from nursing staff, physicians, and certified registered nurse anaesthetists. Positive comments from staff included perceived benefits of SSC for mothers ‘The moms really like having the baby with them’ (Nurse) and for infants ‘The baby is pinker when on the mom's skin instead of in the warmer’ (Physician). Negative comments related to staffing ‘the biggest issue I see is adequate staffing’ (Nurse); time constraints ‘This will not work. I am too fast, and the nurses will not have time to give the baby to the mother before I am done’ (Physician), and lack of engagement with the intervention ‘I am not doing this’ (Anaesthetist).

#### 3.2.3 Mother's experiences

Three studies explored the mother's experiences of the intervention. Haxton et al.^21^ presented two positive anecdotal quotes from mothers who received SSC in the labour and delivery room as part of the intervention, e.g. ‘I forgot about my pain and the baby's color got better’. Two studies examined mothers' experiences of SSC after caesarean section. Boyd^22^ received six comments from mothers, all of which were positive (e.g., ‘I will write a testimony for you as to how good this is’). Similarly, Hung and Berg^13^ received verbal feedback from mothers who stated that they would like to have SSC with their babies in the OR if they were to experience a caesarean again, and commonly concluded that SSC had made them feel happy.

## 4 DISCUSSION

Overall, the available evidence highlights that all interventions had some positive impacts on SSC rates, in terms of increasing the number of women practising SSC and the amount of time mothers were able to be in SSC with their infant. However, while several interventions led to an increased time spent in SSC, this was still for a restricted period of time (typically <15 min with one study reporting the largest period of 42.5 min). Some interventions also did not report any baseline data or utilise inferential statistics^22^, ^25^, ^28^, ^29^ which makes assessing the effects of the intervention and the generalisability of the findings difficult. These data indicate that although there were targeted efforts to increase SSC practices, the results were still not aligned with WHO recommendations or the BFHI standard of immediate and uninterrupted SSC for at least 60 min.^1^, ^35^ It may be that the barriers noted in terms of staff resources (time, volume of workload) and a lack of knowledge and training around the importance of SSC compromise implementation. Given that SSC is underpinned by the nine instinctive stages,^2^ which happen spontaneously in the first hour after birth, it is imperative that interventions aim to meet the 60‐min standard and allow the full potential of newborns instinctive behaviours to occur.

Similarly, while most SSC interventions were found to increase breastfeeding/exclusive breastfeeding rates, inferential statistics were rarely reported,^21^, ^27^ and where present did not meet significance.^24^, ^25^ This could in part be related to the limited duration of SSC as noted above, thus preventing infants from being able to engage in the later stages i.e. suckling. However, as most of these studies focus on improving SSC practices for women following caesarean section,^13^, ^22^, ^25^, ^26^, ^28^, ^29^ there are also additional complications of anaesthesia and recovery to consider that may complicate infant and maternal behaviours post‐delivery, including breastfeeding.^36^ While important, SSC is only one practice that can influence breastfeeding behaviours, with robust evidence of women requiring early and ongoing, proactive, face‐to‐face support from health professionals to improve breastfeeding outcomes.^37^

There was a range of theoretical approaches used to design the interventions, often without a clear rationale for adoption, or consideration of alternative approaches. The most common approach utilised was the Iowa Model (*n* = 5) which is a seven‐stage systematic method that explicates how organisations change practice in order to encourage quality healthcare.^30^ However, whilst this a widely used approach, it has been criticised due to the complexity of the model and difficulty in achieving certain steps in practice.^38^ This was apparent in the current review with one paper failing to provide details on the team recruited to support implementation,^27^ and two failing to evidence that all seven steps had been completed.^22^, ^27^ Other studies used theories which were not specific to practice in a healthcare setting (e.g. Lewin's Change Theory, DMAIC, PDSA). One study used a bespoke quality improvement model (PRECESS) with no reported evidence base,^24^ and three studies did not use any theoretical framework in the development of their interventions.^25^, ^26^, ^29^ Consideration of implementation frameworks such as i‐PARIHS,^39^ and implementation strategies that have a clear evidence‐base, including ERIC^40^ and EPIC,^41^ may help to standardise approaches and to understand the key resources and mechanisms, such as training, self‐efficacy and guidelines, that need to be in place to achieve positive practice change.

Importantly, the majority of studies included in this review were QI projects with small sample sizes and a lack of consistent outcomes was observed. Some of the studies also lacked specific details about the intervention components,^25^ and despite requesting further information from the authors, this was not forthcoming. Where details were provided, there was evidence of multiple, overlapping methods being utilised such as training or education sessions, bulletin board postings, web‐based resources, multidisciplinary teams and nominated champions. Previous research in infant feeding has reinforced the importance of mapping interventions onto behaviour change techniques to see what is driving the effect.^42^ However, the limited number of studies and heterogeneity in intervention design meant that it was impossible to determine which combinations of methods are more likely to influence change. This issue was also exacerbated by a lack of robust evaluation methods, with some studies relying on anecdotal insights as to whether SSC was experienced or not. Thus, the primary mechanisms of effectiveness remain unclear at this time.

Further, SSC interventions are complex in that they are influenced by and enabled at different levels, that is individual, dyadic (mother–infant, staff–mother), staff and the wider health system. Notably, half of the studies only delivered the intervention to one group (i.e. staff),^13^, ^23^, ^24^, ^27^, ^28^ and in studies where mothers were considered alongside staff, the intervention content was limited to written resources about the benefits of SSC only.^13^, ^21^ Therefore, it is important that future studies adopt a holistic approach that considers all of these influences when designing, delivering and evaluating interventions.

With regards to limitations, the present review only included a small number of studies, meaning conclusions must be drawn with caution. Furthermore, it is possible that some results may have been missed, for example, if they incorporated elements outside of the search terms specified. Each of the studies employed bivariate analyses which failed to control for extraneous variables, suggesting that factors outside of the intervention may have played a role in the intervention outcomes or experiences of SSC. In terms of study characteristics, the majority of investigations were undertaken in the USA (*n* = 9) and only one study supplied the mean age of their participants (*M* = 29.3).^24^ Thus, the effects of geographical and societal context, alongside maternal age, on intervention efficacy and experiences cannot reliably be determined. Moreover, as the majority of studies were designed to increase SSC following caesarean birth (*n* = 6), it may be that the results obtained are context specific as the implementation of interventions may be easier within this more controlled environment. Therefore, it is unclear whether interventions employing these components would be effective in improving SSC in other delivery settings. Thus, future research is required to assess whether the content, experiences, or outcomes of SSC interventions differ cross‐culturally and in more diverse samples and birth settings. In addition, poor staff adherence to interventions and training sessions was witnessed,^24^, ^27^, ^28^ alongside a tendency to focus exclusively on staff experiences of the intervention, as opposed to parents' experiences (*n* = 4). As such, potential barriers to implementation and engagement have not been explored, which represents a clear gap for future research to address.

As previously stated, the current data does not allow for replicability due to large inconsistencies in reporting and missing information, including sample sizes, demographic characteristics, and specifics on intervention modality. Thus, it is recommended that future research employ tools such as the TIDieR checklist^43^ to improve reporting of the intervention process and allow for reliable reproduction of the findings. Future research should also endeavour to implement SSC interventions that are in line with best practice guidelines. Finally, the use of a validated tool to inform intervention design, such as the behaviour change wheel, may aid in incorporating the micro, meso and macro levels of influence needed to promote wide‐scale change and to identify the most useful change techniques for increasing SSC.^44^

To conclude, the evidence suggests that current interventions do have a positive impact on the number of women practicing SSC, but while the amount of time spent in SSC increased, this was not in line with WHO recommendations. Further, while most of the interventions were found to increase rates of breastfeeding and exclusive breastfeeding, changes were seldom significant. Parents experiences of SSC interventions were not routinely collected, and so potential barriers to engagement remain unexplored. As the majority of the research employed varying intervention components and multiple techniques, the most effective mechanisms by which SSC interventions operate are unclear. Therefore, given the poor quality of the evidence available and lack of compliance with current best practice recommendations, more robust research is warranted to determine the efficacy of SSC interventions and to identify the behaviour change techniques that can enable consistent improvements.

### AUTHOR CONTRIBUTIONS

Chloe Moran had primary responsibility for conducting database searches, article screening, data extraction, data synthesis, producing an initial draft of the manuscript and preparation of the manuscript for publication. Dr Gill Thomson and Dr Victoria Moran participated in development of the protocol and search strategy for the study and contributed to quality assessments, the writing of the manuscript and review of the final draft. Dr Victoria Fallon contributed in the same ways as Dr Gill Thomson and Dr Victoria Moran and assisted with full‐text screening, data synthesis and supervised the work of Chloe Moran.

### FUNDING INFROMATION

No funding was secured to conduct this research.

## CONFLICT OF INTEREST

None of the authors have any conflicts of interest to disclose.
